# Supplementary material for: A Quantitative Comparison of Two Methods for Higher-Order EEG Microstate Syntax Analysis
Source: Brain Topogr. 2026 Apr 24;39(3):45. doi: 10.1007/s10548-026-01196-5 (PMC13109229; doi:10.1007/s10548-026-01196-5)
Supplement: Supplementary file 1 — Supplementary Material 1 [file 10548_2026_1196_MOESM1_ESM.pdf]

Supplemental Data

Entropy rate (ER) and sample entropy (SE) values for CN, AD, and Markov surrogates

In this section, we provide a more detailed graphical representation of Figure 3, Figure 4, Figure 5, and Figure 6, and we tabulate the numerical ER and SE values (group means) as well as the p values associated with the Wilcoxon and Mann-Whitney tests. All p values were adjusted for multiple comparisons across the six values of k (word length, history length), using the false discovery rate (FDR, Benjamini-Hochberg method). Significant test results ($p < 0.05$) are indicated by downward triangles above the x-axis, as in the main text. The graphical representations show all individual data points (scatter plot), along with an overlaid box-and-whisker plot. Horizontal bars indicate group medians, the box covers the inter-quartile range, and the whiskers extend to both ends of the distribution.

Control group vs. Markov surrogates

A different presentation of the data shown in Figure 3 is shown in Figure S1.

Table S1 represents the data shown in Figure 3 and Figure S1.

Alzheimer's disease group vs. Markov surrogates

A different presentation of the data shown in Figure 4 is shown in Figure S2.

Table S2 represents the data shown in Figure 4 and Figure S2.

Control group vs. Alzheimer's disease group (ER, SE)

A different presentation of the data shown in Figure 5 is shown in Figure S3.

Table S3 represents the data shown in Figure 5 and Figure S3. Note that the Mann-Whitney U test is a rank sum test with a finite number of possible p values, explaining the occurrence of identical p values for multiple values of k.

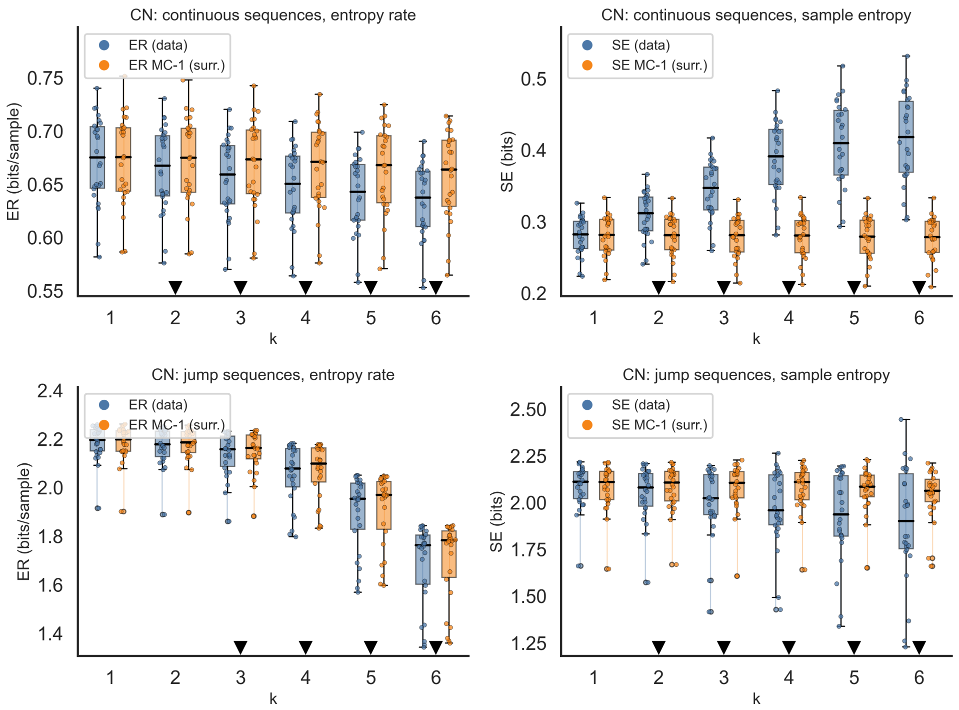

Fig. S1 Entropy rate (ER) and sample entropy (SE) compared for real microstate sequences from the CN group (blue, label: ER/SE (data)) and their first-order Markov surrogates (orange, label: ER/SE MC-1 (surr.)). Statistically significant differences ($p < 0.05$) between real microstate sequences and first-order Markov surrogates are indicated by black downward triangles.

Control group vs. Alzheimer's disease group (normalized syntax measures σ)

A different presentation of the data shown in Figure 6 is shown in Figure S4.

Table S4 represents the data shown in Figure 6 and Figure S4. Note that the Mann-Whitney U test is a rank sum test with a finite number of possible p values, explaining the occurrence of identical p values for multiple values of k.

Alternative EEG pre-processing and cluster numbers

This section illustrates that the results presented in the main text can be reproduced when a different pre-processing strategy is applied to the raw EEG data, and for

Table S1 ER and SE group mean values for the CN group and its first-order Markov surrogates (Markov-1), p values are FDR-corrected results of Wilcoxon tests for two dependent samples, k indicates the microstate word length.

k	CN	Markov-1	p-value
Continuous sequences, entropy rate (ER)			
1	0.550	0.550	0.819
2	0.544	0.550	0.000
3	0.537	0.549	0.000
4	0.530	0.549	0.000
5	0.525	0.548	0.000
6	0.521	0.546	0.000
Jump sequences, entropy rate (ER)			
1	1.522	1.521	0.689
2	1.516	1.521	0.016
3	1.510	1.519	0.002
4	1.504	1.516	0.000
5	1.493	1.508	0.000
6	1.470	1.487	0.000
Continuous sequences, sample entropy (SE)			
1	0.239	0.239	1.000
2	0.260	0.239	0.000
3	0.284	0.238	0.000
4	0.312	0.236	0.000
5	0.319	0.236	0.000
6	0.319	0.235	0.000
Jump sequences, sample entropy (SE)			
1	1.467	1.466	0.587
2	1.452	1.465	0.002
3	1.418	1.464	0.000
4	1.401	1.464	0.000
5	1.384	1.465	0.000
6	1.358	1.465	0.000

different cluster numbers $K = 4, 5, 6$. We pre-processed the raw data in (Miltiadous et al, 2023) without using the Artefact Subspace Reconstruction (ASR) algorithm for ocular artefact removal (Kothe and Makeig, 2013) that was used in the publicly available pre-processed dataset. The ASR algorithm contains free parameters that need to be chosen by the researcher, and these parameters influence to what extent artefact-related features remain in the data, or vice versa, neural signals are removed. We therefore implemented the following ASR-free pre-processing pipeline in MNE

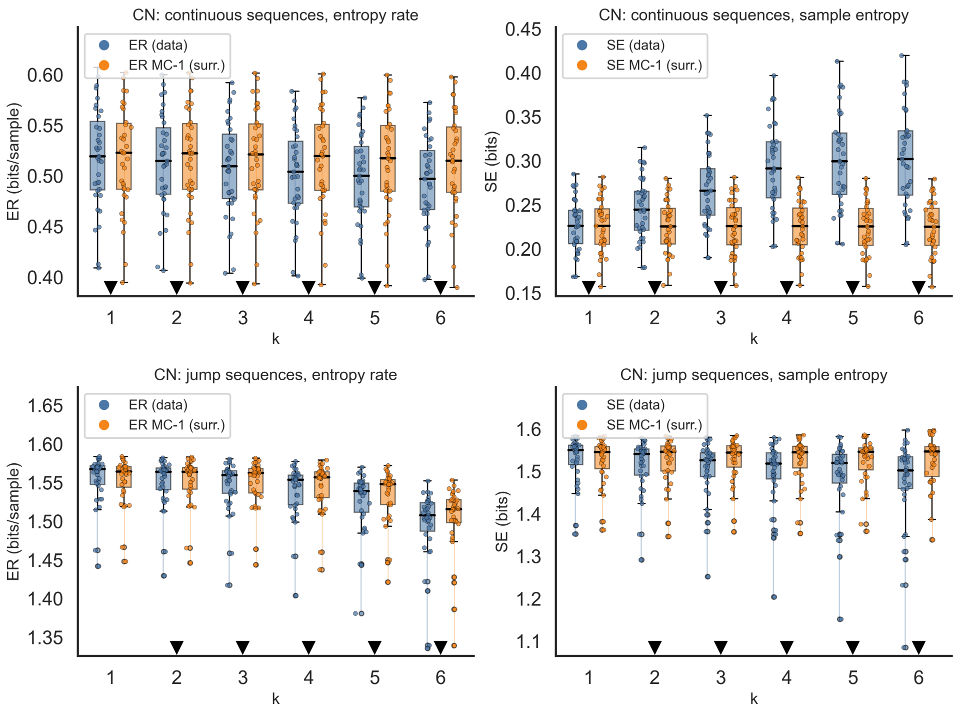

Fig. S2 Entropy rate (ER) and sample entropy (SE) compared for real microstate sequences from the AD group (blue, label: ER/SE (data)) and their first-order Markov surrogates (orange, label: ER/SE MC-1 (surr.)). Statistically significant differences ($p < 0.05$) between real microstate sequences and first-order Markov surrogates are indicated by black downward triangles.

(Gramfort, 2013): (i) exclusion of bad electrodes and EEG epochs with very large artefacts, (ii) ICA, followed by exclusion of components clearly related to vertical or horizontal eye movements, ECG, pulse, muscle or movement artefacts, followed by reconstruction from the remaining components, (iii) interpolation of the signal from electrodes removed in the first step, (iv) final marking of artefact-rich intervals. All other parameters (band-pass filtering, average reference, microstate clustering, back-fitting and entropy calculations) were identical to the procedures described in the main text. Again, only one data segment per subject (10^5 samples) was chosen. To test the influence of the cluster number (K), we performed analyses for $K = 4, 5, 6$ microstate classes. Below, we present the results corresponding to Figure 3 and Figures 4 in the main text. It should be noted that, while the overall shape of the entropy curves (ER,

Table S2 ER and SE group mean values for the AD group and its first-order Marov surrogates (Markov-1), p values are FDR-corrected results of Wilcoxon tests for two dependent samples, k indicates the microstate word length.

k	AD	Markov-1	p-value
Continuous sequences, entropy rate (ER)			
1	0.507	0.508	0.211
2	0.502	0.508	0.000
3	0.497	0.508	0.000
4	0.492	0.507	0.000
5	0.488	0.506	0.000
6	0.485	0.505	0.000
Jump sequences, entropy rate (ER)			
1	1.550	1.549	0.044
2	1.546	1.548	0.058
3	1.542	1.546	0.004
4	1.537	1.542	0.001
5	1.526	1.533	0.001
6	1.500	1.508	0.001
Continuous sequences, sample entropy (SE)			
1	0.219	0.220	0.527
2	0.236	0.219	0.000
3	0.257	0.218	0.000
4	0.280	0.218	0.000
5	0.286	0.217	0.000
6	0.286	0.217	0.000
Jump sequences, sample entropy (SE)			
1	1.520	1.517	0.040
2	1.504	1.517	0.000
3	1.490	1.517	0.000
4	1.484	1.515	0.000
5	1.479	1.517	0.000
6	1.469	1.517	0.000

SE) is similar, absolute entropy values change with K, since the maximum entropy values scales as $\log(K)$.

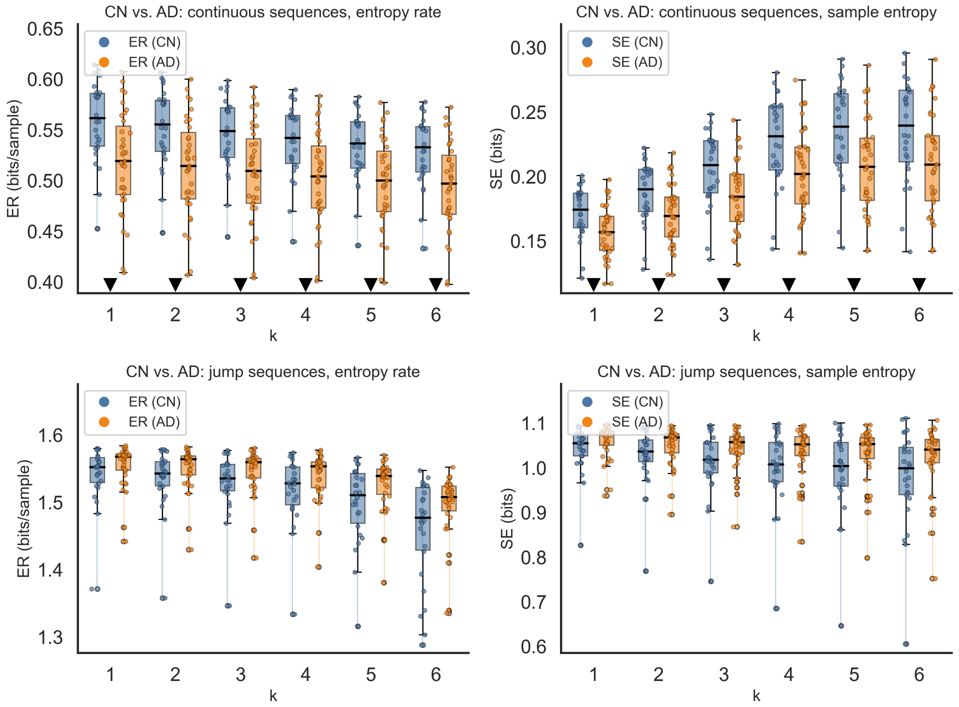

Fig. S3 Direct comparison of CN (blue) and AD (orange) subjects. Statistically significant differences ($p < 0.05$, black downward triangles) were found for all tested syntax levels ($k = 1 - 6$), for continuous and jump microstate sequences, and for both syntax metrics (ER and SE).

Table S3 ER and SE group mean values for the CN and AD groups, p values are FDR-corrected results of Mann-Whitney U tests for two independent samples, k indicates the microstate word length.

k	CN	AD	p-value
Continuous sequences, entropy rate (ER)			
1	0.550	0.507	0.002
2	0.544	0.502	0.002
3	0.537	0.497	0.002
4	0.530	0.492	0.002
5	0.525	0.488	0.002
6	0.521	0.485	0.002
Jump sequences, entropy rate (ER)			
1	1.522	1.550	0.007
2	1.516	1.546	0.007
3	1.510	1.542	0.007
4	1.504	1.537	0.007
5	1.493	1.526	0.007
6	1.470	1.500	0.018
Continuous sequences, sample entropy (SE)			
1	0.239	0.219	0.014
2	0.260	0.236	0.014
3	0.284	0.257	0.014
4	0.312	0.280	0.014
5	0.319	0.286	0.014
6	0.319	0.286	0.014
Jump sequences, sample entropy (SE)			
1	1.467	1.520	0.007
2	1.452	1.504	0.008
3	1.418	1.490	0.008
4	1.401	1.484	0.005
5	1.384	1.479	0.005
6	1.358	1.469	0.005

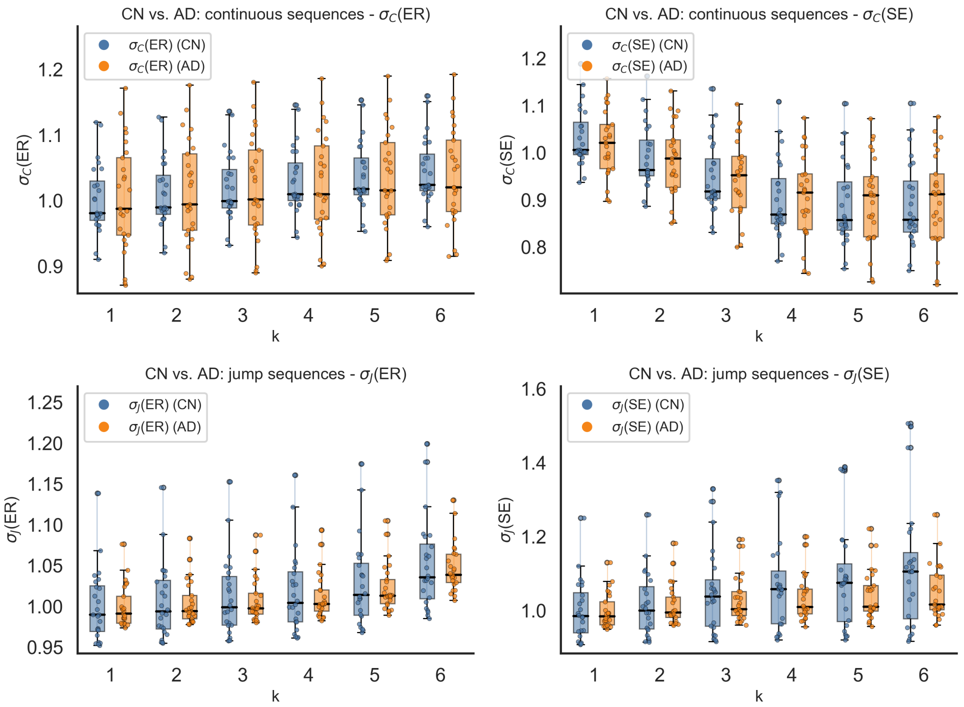

Fig. S4 Normalized syntax measures σ_C and σ_J applied to ER (left) and SE (right) for CN (blue) and AD (orange) groups. No statistically significant differences were found between CN and AD groups in continuous or jump microstate sequences.

Table S4 Normalized syntax measures σ_C and σ_J applied to ER and SE (group means) for the CN vs AD groups, p values are FDR-corrected results of Mann-Whitney U tests for two independent samples, k indicates the microstate word length.

k	CN	AD	p-value
Continuous sequences, entropy rate (ER)			
1	1.002	1.004	0.496
2	1.011	1.011	0.496
3	1.020	1.019	0.496
4	1.031	1.027	0.496
5	1.039	1.033	0.496
6	1.045	1.037	0.496
Jump sequences, entropy rate (ER)			
1	1.000	1.000	0.425
2	1.006	1.004	0.425
3	1.011	1.008	0.425
4	1.018	1.013	0.425
5	1.028	1.023	0.425
6	1.051	1.047	0.425
Continuous sequences, sample entropy (SE)			
1	1.032	1.021	0.456
2	0.992	0.986	0.456
3	0.948	0.948	0.456
4	0.901	0.908	0.456
5	0.890	0.899	0.456
6	0.890	0.900	0.456
Jump sequences, sample entropy (SE)			
1	1.000	0.999	0.348
2	1.015	1.014	0.348
3	1.051	1.028	0.348
4	1.069	1.034	0.348
5	1.087	1.039	0.348
6	1.117	1.049	0.348

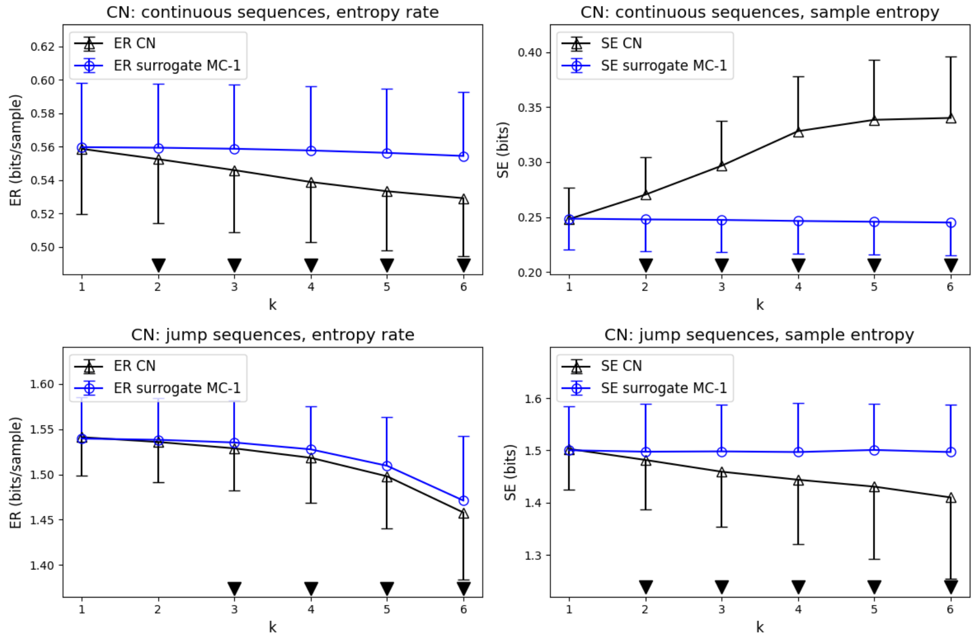

Fig. S5 EEG pre-processing **without ASR for K=4** microstate classes. Entropy rate (ER) and sample entropy (SE) compared for real microstate sequences from the **CN group** (black) and their first-order Markov surrogates (blue, label contains 'surrogate MC-1'). Statistically significant differences ($p < 0.05$) between real microstate sequences and first-order Markov surrogates are indicated by black downward triangles.

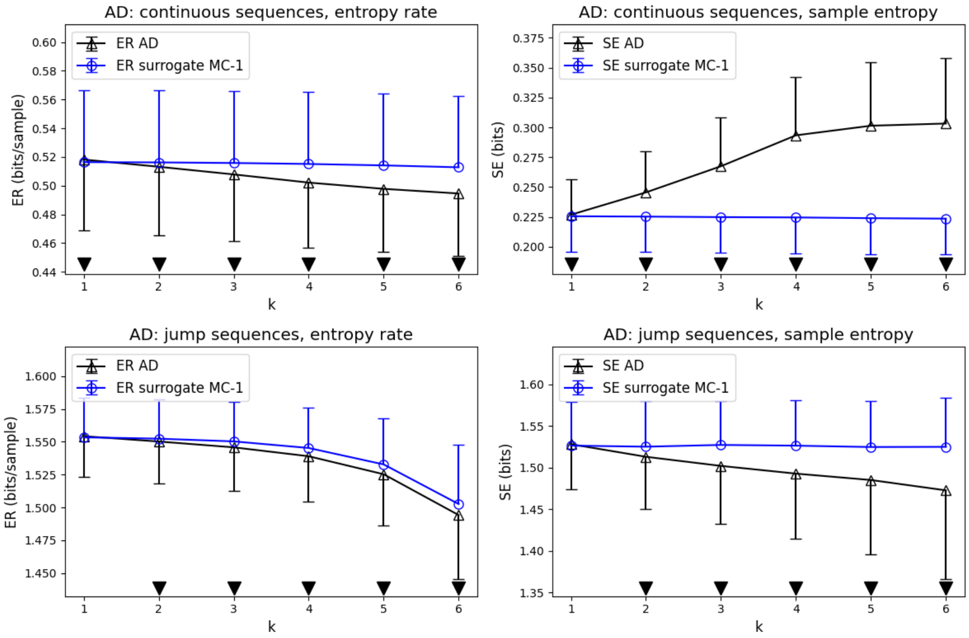

Fig. S6 EEG pre-processing **without ASR for K=4** microstate classes. Entropy rate (ER) and sample entropy (SE) compared for real microstate sequences from the **AD group** (black) and their first-order Markov surrogates (blue, label contains 'surrogate MC-1'). Statistically significant differences ($p < 0.05$) between real microstate sequences and first-order Markov surrogates are indicated by black downward triangles.

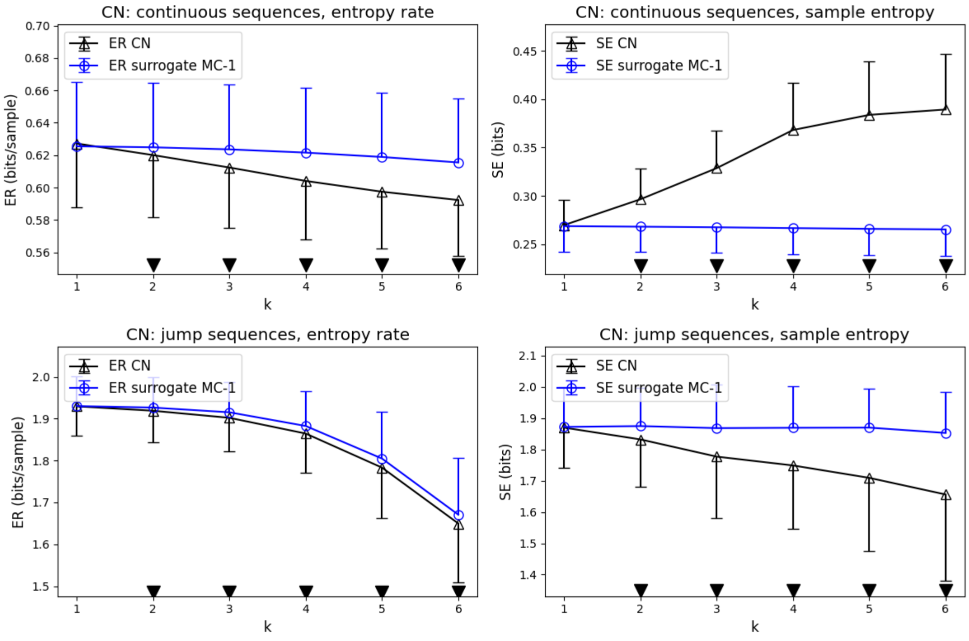

Fig. S7 EEG pre-processing **without ASR for K=5** microstate classes. Entropy rate (ER) and sample entropy (SE) compared for real microstate sequences from the **CN group** (black) and their first-order Markov surrogates (blue, label contains 'surrogate MC-1'). Statistically significant differences ($p < 0.05$) between real microstate sequences and first-order Markov surrogates are indicated by black downward triangles.

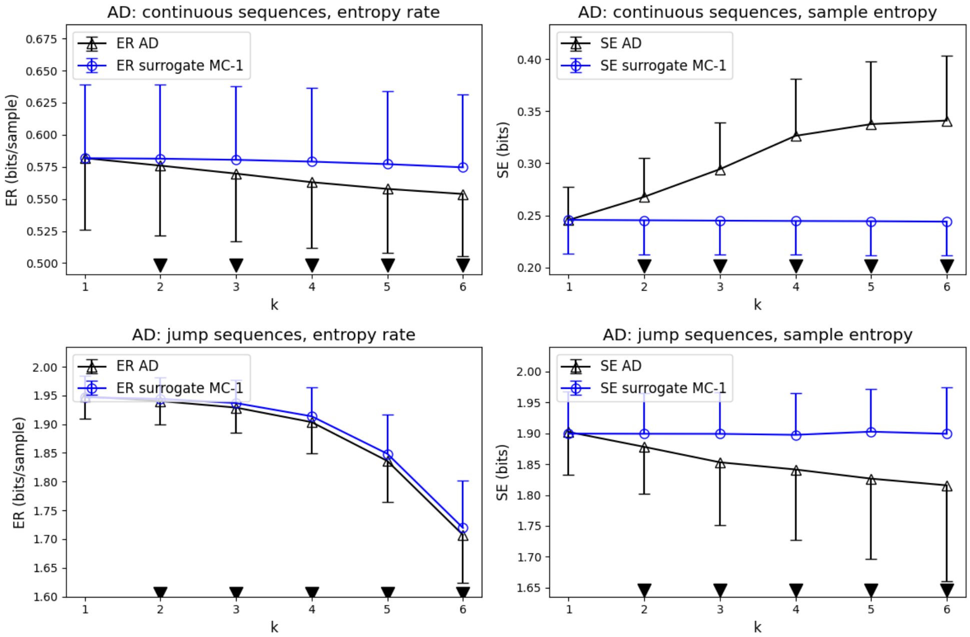

Fig. S8 EEG pre-processing **without ASR for K=5** microstate classes. Entropy rate (ER) and sample entropy (SE) compared for real microstate sequences from the **AD group** (black) and their first-order Markov surrogates (blue, label contains 'surrogate MC-1'). Statistically significant differences ($p < 0.05$) between real microstate sequences and first-order Markov surrogates are indicated by black downward triangles.

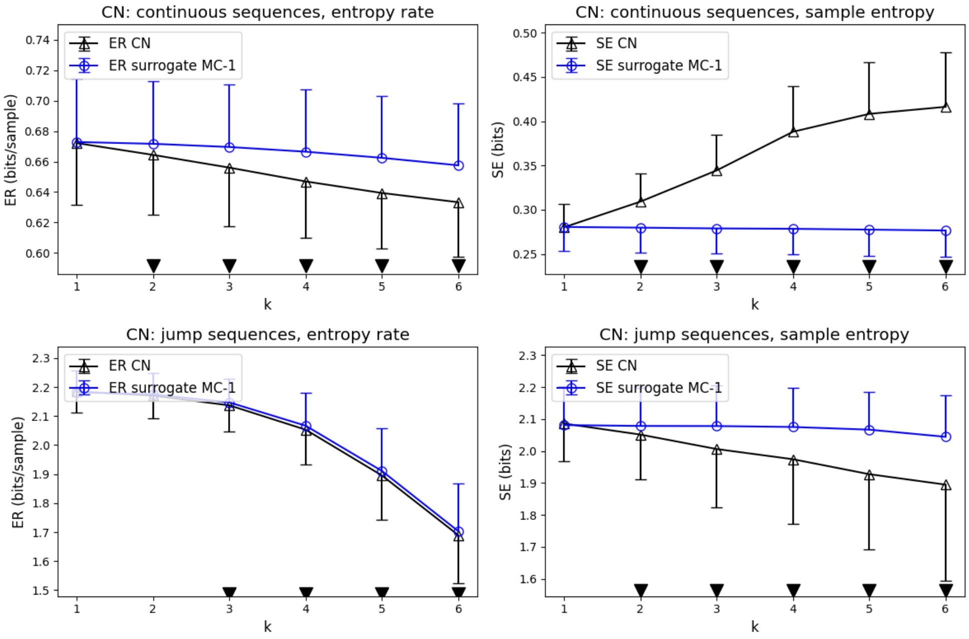

Fig. S9 EEG pre-processing **without ASR for K=6** microstate classes. Entropy rate (ER) and sample entropy (SE) compared for real microstate sequences from the **CN group** (black) and their first-order Markov surrogates (blue, label contains 'surrogate MC-1'). Statistically significant differences ($p < 0.05$) between real microstate sequences and first-order Markov surrogates are indicated by black downward triangles.

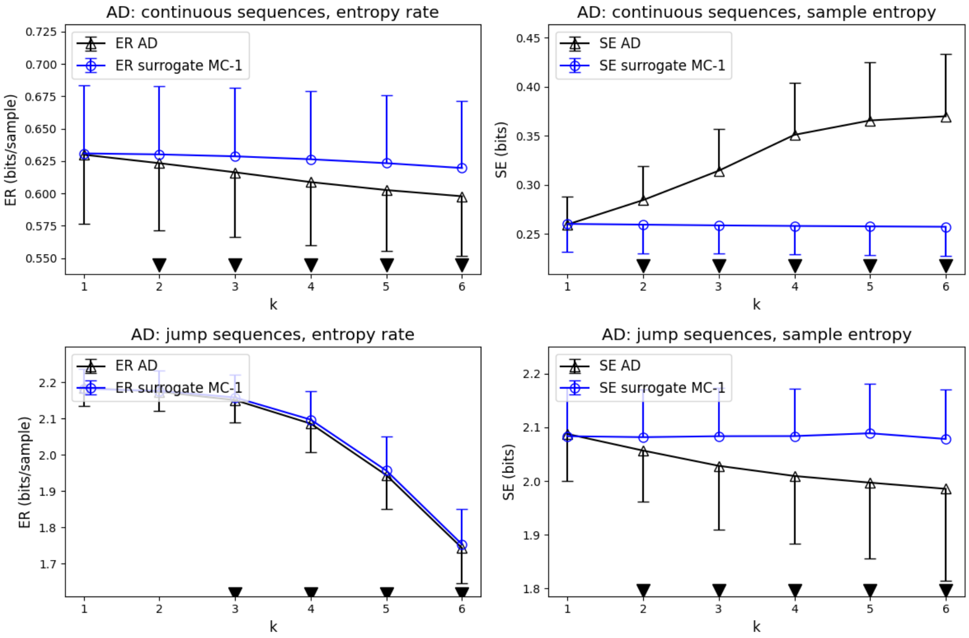

Fig. S10 EEG pre-processing **without ASR for K=6** microstate classes. Entropy rate (ER) and sample entropy (SE) compared for real microstate sequences from the **AD group** (black) and their first-order Markov surrogates (blue, label contains 'surrogate MC-1'). Statistically significant differences ($p < 0.05$) between real microstate sequences and first-order Markov surrogates are indicated by black downward triangles.
